# A Mixed-Method Examination of Emerging and Young Adult Cancer Caregivers’ Experiences during the COVID-19 Pandemic

**DOI:** 10.3390/ijerph20085537

**Published:** 2023-04-17

**Authors:** Amanda Kastrinos, Kelsey Bacharz, Emily L. Mroz, Carla L. Fisher, Allison J. Applebaum

**Affiliations:** 1 Memorial Sloan Kettering Cancer Center, Department of Psychiatry and Behavioral Sciences, New York, NY 10065, USA; 2 Department of Clinical & Health Psychology, University of Florida, Gainesville, FL 32601, USA; 3 Department of Internal Medicine, Yale School of Medicine, New Haven, CT 06520, USA; 4 University of Florida Health Cancer Center, Gainesville, FL 32601, USA

**Keywords:** caregiving, cancer, COVID-19, young adults

## Abstract

Advanced cancer caregivers in emerging and young adulthood (EYACs; ages 18–35) are an understudied yet vulnerable caregiving population. The COVID-19 pandemic created new challenges for advanced cancer caregivers but also created unique contexts from which caregivers sometimes benefited. To understand how the pandemic may have positively and negatively impacted their caregiving and bereavement experiences, we examined EYACs’ experiences of caring for and losing a parent with advanced cancer during the pandemic in comparison to those of EYACs with a parent who died outside the context of the pandemic. Eligible EYACs completed an online survey and semi-structured interview. Quantitative analyses compared responses for pre-pandemic EYACS (*n* = 14) and pandemic EYACs (*n* = 26). A thematic analysis of the interview transcripts of pandemic EYACS (*n* = 14) was conducted. Pandemic EYACs experienced non-significant but higher communal coping, benefit finding, negative emotional experiences, and caregiver strain than pre-pandemic EYACs. Thematic analysis revealed that the pandemic negatively affected EYACs’ caregiving efficacy, personal well-being, interpersonal dynamics, and bereavement; shifts to remote work and schooling were reported as benefits. The findings can inform the design of resources to support EYACs whose parents died during the pandemic and who are navigating the healthcare system today.

## 1. Introduction

The COVID-19 pandemic created massive disruptions to the global healthcare system, the long-term impacts of which are still unknown. One group particularly vulnerable to these disruptions were family members caring for a loved one with cancer. Over half of all family caregivers in the United States (U.S.) experienced significant changes to their caregiving arrangements due to the pandemic [1]. Beginning in March 2020, the U.S instituted social distancing guidelines that encouraged the public to stay at home, limited in-person contact among healthcare providers and patients, and barred family caregivers from visiting their loved ones in the hospital or other care facilities [2,3]. Many cancer caregivers were forced to navigate the loss of in-person support, restrictions on mobility due to mandated lockdowns and physical distancing requirements, and pervasive fear over their loved ones’ COVID-19 infection risk [1,4]. Scholars have called for research that identifies the specific psychological and social needs of subgroups of cancer patients and caregivers as a result of the pandemic [5,6].

Cancer caregiving is extremely challenging even in a pre-pandemic world, with caregivers facing significant physical, psychosocial, and financial burdens [7,8]. The strain that the COVID-19 virus imparted on the healthcare system left cancer caregivers worldwide shouldering additional burdens and taking on more responsibility in the delivery of their loved ones’ care [5]. These impacts are especially salient for caregivers of patients with advanced cancer, for whom the rapidity of their disease progression heightens the scope and urgency of the responsibilities associated with their care [9,10,11]. Thus, it is likely that caregivers of patients with advanced cancer may have been uniquely affected by the COVID-19 pandemic and therefore at a higher risk for experiencing psychological distress.

Emerging and young adult caregivers (EYACs) are understudied in cancer caregiving scholarship, and they face a distinct set of challenges based on their age and developmental stage. Although cancer caregivers tend to be middle-aged or older adults [12], there is a growing group of caregivers in young adulthood. Caregivers between 18 and 30 now account for 43% of all U.S. caregivers, and many provide care to a parent or parent-in-law [11,12]. EYACs experience distinct barriers to, and consequences of, parental caregiving; as a result, they report pervasive psychosocial support needs. A recent systematic review found having a parent with cancer during adolescence or young adulthood is linked to depression, anxiety, stress, worry, and post-traumatic distress [13]. When compared to both older adult caregivers and similarly aged non-caregivers, EYACs report higher rates of anxiety and depression, and the impact of caregiving so early in their lifespan can have developmental and psychological implications that persist long after their caregiving role has ended [7,14,15]. The responsibilities of caregiving often derail EYACs’ pursuits of higher education, romantic relationships, or career advancement, leaving them feeling isolated and out of step from their peers [16,17]. Despite these consequences, there are limited resources available to support EYACs’ psychosocial health.

One issue EYACs often face when caring for a parent with cancer is a lack of information sharing within their family. Parents diagnosed with cancer are known to withhold information about their illness from their adult children in an attempt to reduce their distress, despite relying on them to engage in care tasks and support the navigation of disease progression [18,19]. This fragmented flow of information can limit EYACs’ ability to engage in communal coping, an interpersonal process where coping resources are shared between caregivers and close contacts (e.g., the patient, family members). Communal coping is linked to better psychosocial health outcomes for patients and family caregivers in a number of different disease contexts [20,21,22]. Given that EYACs are more likely to rely on maladaptive coping strategies than older adult caregivers, their need for communal coping is even greater. This is especially true for EYACs of a parent with advanced cancer, as the accelerated disease timeline amplifies the risks of psychological distress [9,10].

Considering the additional burdens placed on cancer caregivers during the COVID-19 pandemic, it is likely that EYACs who cared for and lost a parent to advanced cancer during this period experienced significant psychosocial impacts that are still salient after their parents’ death. While negative impacts are probable, several studies of pandemic caregivers in other age groups identified a number of perceived benefits of caregiving during the pandemic [18], including perceived improvements in caregivers’ health behaviors, spirituality, and family relationships and communication [18,19]. The purpose of this study was to examine how the COVID-19 pandemic affected EYACs’ experiences of caring for and losing a parent with advanced cancer and to identify differences from the experiences of EYACs with a parent who died before the start of the pandemic. We employed a mixed-method approach to: (1) quantitatively compare the experiences of EYACs who provided care before the pandemic with those who provided care during the pandemic, examining potential differences in their communal coping with their family members as well as differences in secondary outcomes of caregiver strain, emotional experiences, and benefit finding; and (2) qualitatively examine perceived positive and negative impacts of the pandemic on EYACs’ caregiving and loss experiences.

## 2. Materials and Methods

### 2.1. Participants

Data were collected as part of a larger mixed-method study examining EYACs’ caregiving and coping experiences in the advanced cancer context. Purposive sampling was used to recruit EYACs using the social media site Reddit and ResearchMatch, a U.S. national health registry that has a large population of volunteers who have agreed to be contacted by researchers about health studies. Messages were sent via ResearchMatch to over 50,000 randomly selected volunteers between the ages of 18 and 35 over the course of three months. Study advertisements were also posted to cancer, caregiving, and grief forums (i.e., subreddits) on Reddit.

To be eligible for the study, participants: (1) were between the ages of 18–35; (2) served as a caregiver for a parent with cancer; (3) were bereaved between March 2020 and the time of data collection (November–December 2021); and (4) their parent’s cancer trajectory from diagnosis to death was less than 12 months. Forty participants completed the online survey, and 33 of those went on to complete an interview regarding their caregiving experience. Of those 33, 14 EYACs were caregivers during the COVID-19 pandemic and were thus included in the qualitative analysis.

### 2.2. Measures

*Demographic characteristics.* Participants were asked questions about their age, gender, race, education, and their parents’ cancer type. For descriptive purposes, they also completed the 31-item Inventory of the Dimensions of Emerging Adulthood (IDEA) to better characterize their developmental stage. The IDEA measures] five dimensions that reflect emerging adulthood: identity exploration, experimentation/possibilities, negativity/possibility, self-focused, and feeling in-between [23]. The scale asks participants questions such as: “Is this period of your life a time of personal freedom?” Responses range from 1 (*strongly agree*) to 4 (*strongly disagree*).

*Communal Coping.* The 15-item Communal Coping Scale measures participants’ engagement in interpersonal coping strategies [24]. Participants were asked the extent to which they engaged in specific coping activities with their family during their parents’ cancer on a scale from 0 (*never*) to 4 (*always*) (α = 0.81). The wording on some items was modified to fit the cancer context. Sample statements included “We have told or expressed to one another how we feel” and “We have tried to keep our emotions to ourselves and do not show them in front of others.”

*Positive and Negative Elements of Parental Cancer.* The Parental Cancer Questionnaire (PCQ) measures participants’ perceived benefits, negative emotional impact, and strain of caregiving for a parent with cancer [25]. The scale has 33 items and utilizes a 5-point Likert-type scale (1 = *strongly disagree* and 5 = *strongly agree*) (α = 0.83). The scale is made up of three subscales: 16 items measuring caregivers’ perceived benefits (e.g., “I became closer to my sick parent during his/her cancer”; α = 0.72); 10 items measuring their emotional reaction to their parent’s cancer (e.g., “I feel great sadness when I think about my parent’s cancer”; α = 0.78); and 7 items measuring caregiver strain (e.g., “I felt angry that my life was changed because of my parent’s cancer”; α = 0.86). The wording on some items was adapted to reflect their bereavement.

### 2.3. Procedure

Potential volunteers were sent a link to an online screening survey hosted by REDCap in which they provided information to determine their eligibility. We contacted eligible participants by phone or email to schedule a phone or video interview. Prior to the interview, we asked participants to complete the above measures via an online survey. Interviews were conducted using the Retrospective Interviewing Technique (RIT), a widely used in-depth lifespan interviewing technique that captured retrospective data about EYACs’ caregiving experiences across their parent’s disease trajectory [26,27].

We asked EYACs who were caregivers during the pandemic specific questions regarding its impact on their caregiving, bereavement, memorialization, and adjustment experiences. Sample questions included “Do you think COVID-19 played a role in your caregiving experience?” and “Did COVID-19 impact your ability to grieve after the loss of your parent? If yes, how?” The interviews were audio recorded and transcribed, resulting in 817 pages of data. The length of interviews ranged from 40 to 113 min. Participants received $35 as compensation upon completion of the survey and interview. These procedures were approved by the Institutional Review Board of a large southeastern university (IRB202001910).

### 2.4. Analysis Plan

To address our quantitative aim, survey data were downloaded, cleaned, and analyzed via SPSS v.27 software. The sum or average of each measure was calculated depending on the specific scoring guidelines. All participants who completed the measures were included in the quantitative analyses, regardless of whether or not they completed the interview. We created a grouping variable to distinguish whether the participant was a caregiver prior to or during the COVID-19 pandemic. We calculated descriptive statistics and frequencies of the demographic features of the full sample and within each group. We conducted independent *t*-tests assessing for group differences based on the timing of the caregiving (i.e., pre-pandemic and pandemic caregivers) in relation to communal coping and the three PCQ subscales (i.e., benefit finding, emotional experience, and caregiver strain). Both the *p*-value and effect size were reported, but given the small sample size, the results were primarily interpreted based on the effect size, which is less impacted by sample size [28]. While reporting the *p*-value is often utilized as a measure of statistical significance, reporting the effect size is often beneficial for articulating the practical significance of the results. There are multiple types of effect sizes that can be calculated. We chose to report Hedges’ g, which is a type of effect size calculation that corrects for small sample size, with 0.20, 0.50, and 0.80 indicating small, medium, and large effect sizes, respectively [29,30]. A larger effect size indicates that the result is more practically significant.

To address our qualitative aim, we analyzed interview transcripts using ATLAS.Ti software to manage the data. Two authors concurrently conducted a thematic analysis of the interview transcripts using a constant comparative approach [31,32,33]. In order to highlight the unique experience of caring for a parent with advanced cancer during the COVID-19 pandemic, we only analyzed the transcripts of pandemic caregivers (*n =* 14), excluding pre-pandemic caregivers. The data were analyzed using the following steps: (1) identify concepts within the transcripts and assign them codes; (2) use the criteria for thematic saturation (repetition, reoccurrence, and forcefulness) to collapse the codes into categories (i.e., themes); (3) axial code each category to determine its thematic properties, ensuring a rich description; and (4) identify relationships between the themes. The two authors held consensus meetings regularly throughout the coding process to maintain agreement. This process resulted in a codebook that included themes, properties, descriptions, and exemplar participant quotes. The themes and thematic properties were then validated by a third author who used the codebook to analyze a subset of the transcripts, demonstrating that the codebook captured salient and consistent themes.

## 3. Results

### 3.1. Participants

Participants (*N* = 40) were primarily female, White, Non-Hispanic college graduates who cared for a father, had at least one sibling, and their parents were married. The demographic variables are presented below (Table 1) and divided into the timing of their caregiving experience (i.e., prior to or during the COVID-19 pandemic).

### 3.2. Quantitative Findings

The independent *t*-test examining group differences in communal coping demonstrated a non-significant, medium effect of group; pandemic caregivers reported higher levels of communal coping than pre-pandemic caregivers, t(37) = 1.88, *p* = 0.07, g = 0.59.

The three *t*-tests looking at group differences in PCQ subscales were also not significant according to *p*-values; however, they indicated small effects of group on each outcome. Specifically, pandemic caregivers reported higher benefit finding [t(24) = 0.57, *p* = 0.57, g = 0.23], higher negative emotional experiences [t(35) = 0.80, *p* = 0.43, g = 0.26], and higher caregiver strain [t(31) = 0.92, *p* = 0.36, g = 0.32] than pre-pandemic caregivers. Means and standard deviations of the outcome variables by group are shown in Table 2. Notably, the standard deviations appeared to be large, which can be an indication of variability in participant responses. However, statistical tests of normality and homogeneity of variance, which are also measures of variability in the data, indicated that, overall, the variance in the data was not statistically significant. Thus, it is likely that the high standard deviations were due to the small sample size.

### 3.3. Qualitative Findings

EYACs described both positive and negative impacts on their caregiving and bereavement experiences as a result of the COVID-19 pandemic. Four themes were identified encompassing these impacts: (1) perceived quality of their parent’s care; (2) caregiving efficacy; (3) personal and interpersonal dynamics; and (4) remembrance services and bereavement. These themes are presented below with thematic properties presented in italics to further define each theme. Participant narratives illustrate each of these impacts. Participants are identified in the exemplar quotes below by their age and relationship to the patient. Nearly all caregivers cared for their parent both at home and in the hospital at various points in their disease trajectory.

#### 3.3.1. Perceived Impacts to Their Parents’ Care

EYACs felt that the stress of the COVID-19 pandemic on the medical system impacted the cancer care their parent received. For example, EYACs reported believing that the pandemic led to their parent receiving *delayed cancer diagnoses.* Fears over exposure to the COVID-19 virus at the hospital kept their parent from seeking care for emerging cancer symptoms, thus delaying the discovery of their cancer. Others felt the influx of COVID-19 patients pushed their parents’ cancer lower on clinicians’ priority list. One daughter believed this delay may have led to her father receiving a more advanced prognosis at diagnosis:

*Back in February, he actually had a scan, and they actually saw the nodes … They were concerned about it, and he needed to do something to get checked out. COVID hit in March … I think that because the doctors werent banging down the door telling him that it was cancer he just didnt do anything from February until July. By then it had metastasized all over.*

*(Daughter, 34)*

EYACs also described feeling like their parent *received lower quality care* than if the pandemic had not occurred. For instance, EYACs reported their parent receiving some, or all, of their cancer care via telehealth, which one daughter described as “completely unacceptable” (Daughter, 25). They recalled experiencing delays in testing and difficulty making medical appointments, as well as logistical challenges that stemmed from having to demonstrate negative COVID-19 tests before each appointment or procedure. One daughter explained the added stress of these delays on her family:

*[Dad] started getting bronchitis, and due to the fact that he had a cough, which is one of the symptoms of COVID, [clinicians] freaked out pretty badly. [They] kicked him out of his appointment and canceled all of his future appointments.*

*(Daughter, 21)*

These challenges were an extra burden to their already difficult caregiving experiences, and EYACs described feeling emotional distress over the way their parent was treated. This daughter later explained: “[The pandemic] kind of delayed everything, and it’s kind of left me wondering if maybe they tried to treat his bronchitis sooner, he would still have a year that the doctors originally predicted that he would have” (Daughter, 21).

#### 3.3.2. Caregiving Efficacy

At times, EYACs felt that the pandemic made it easier to be an effective caregiver for their parent. They reported that *employment changes due to the pandemic enabled them to become caregivers* for their diagnosed parent when ordinarily they may not have had the opportunity due to living arrangements or responsibilities. Furloughs or shifts to remote work and schooling allowed some EYACs to move into the same house as their diagnosed parent. Given that many were previously living states, or even countries, away before the start of the pandemic, this move gave EYACs a unique opportunity to step into the caregiving role for their parent. Some EYACs described being grateful for the circumstances that allowed them to provide care to their parent, as this daughter explained: “[My mother] needed a lot of help. And so, we would always be right next to her. So, I was pretty happy that we were in quarantine, so I would be able to help her and help my dad out” (Daughter, 26).

Other EYACs felt the pandemic restricted their caregiving efficacy. They recalled the pain of *being unable to visit their parent in the hospital or accompany them to appointments*. This limited their ability to serve as an advocate for their parent or stay informed about their prognosis and care, as this son described:

*I couldn’t get into the hospital. I wanted to side with the doctors, but I also didn’t really understand why she was in the hospital as long as she was … It was just difficult to say, “Mom you’ve been there for over a week, and I’m sure it’s the right thing to do but I can’t tell you why.” So, yeah, that was a very challenging point.*

*(Son, 22)*

These limitations had an increasingly negative impact as their parents’ cancer progressed and they transitioned to end-of-life care. Not only were EYACs left feeling helpless, as they were unable to be present and provide support to their dying parent in the hospital, but the separation also created substantial emotional distress:

*It was a lot of struggle … The nurse would put an iPod in front of her, and we would call occasionally to make sure she didn’t feel alone … She wouldn’t really respond to us. She couldn’t say anything back … There was a lot of crying because we thought she was going to die.*

*(Son, 19)*

In addition to the separation from their parent, EYACs reported that their caregiving efficacy was affected by the *lack of in-person family support.* Travel restrictions and fears over infecting their immunocompromised parent meant EYACs had greater caregiving demands and a smaller in-person support system. This was especially difficult for EYACs living alone with their diagnosed parent. At times EYACs reported feeling lost and overwhelmed with their caregiving responsibilities, and these feelings were exacerbated by the knowledge that they would have had help from family members if not for pandemic restrictions. One daughter, whose family lived outside of the country and were unable to come to the U.S., described:

*It was really hard. Because I wanted them to just come, which I knew they would if it was in normal circumstances … At the time I was little frustrated. Like—because I was trying—I called the Department of Homeland Security, and I asked them if they could make an exception for my aunt to come.*

*(Daughter, 30)*

#### 3.3.3. Personal and Interpersonal Dynamics

EYACs reported that the pandemic had both positive and negative impacts on their personal well-being and interpersonal relationships. For example, EYACs were able to *spend quality time at home with their parent* in a way they would not have been able to if not for the pandemic, which they described positively. Employment changes, as well as the cancellation of travel and other activities, gave some EYACs a unique opportunity to spend time at home with their families. One daughter explained how this increased time affected her after her father’s death: “The COVID situation has actually massively helped me be able to spend time with him and have that time to grieve while my dad was alive, but also to be involved and to get those last few months with him” (Daughter, 34).

But spending time with their parent also created additional fears for EYACs, as they struggled with a persistent fear of infecting their parent with the COVID-19 virus. For those living outside the home, this fear limited the number of in-person visits they had with their diagnosed parent. For those living with their diagnosed parent, this fear limited their involvement in activities outside the home that may have helped them maintain a sense of normalcy. In both cases, EYACs described added stress and anxiety over their parents’ risk of exposure. This son explained:

*It made me paranoid as shit because I’m afraid to touch any little thing outside in public or be around just strangers … I think if COVID hadn’t been a thing I still would obviously be in a stressful situation, but you don’t have that layer on top of it.*

*(Son, 22)*

EYACs also experienced heightened stress as a result of *sacrificing self-care* due to pandemic restrictions. Activities that EYACs might have typically engaged in outside of caregiving to take their mind off their parents’ stressful situation, such as exercise, work, or spending time with friends, were now unavailable. They described how their inability to participate in typical daily routines or take time away from home to relieve their stress greatly impaired both their well-being and that of their diagnosed parent:

*It was pretty lonely. I couldn’t really go anywhere. I’m a huge retail therapy person, and I couldn’t go shopping. I couldn’t do anything. And my dad couldn’t go to the grocery store, like anything that would make him feel like normal.*

*(Daughter, 23)*

Finally, EYACs reported that concerns over their parent’s COVID-19 exposure made them feel like they were *parenting their parent.* They described how differing levels of concern within their family over how to manage their parents’ risks of exposure forced them to take a “parental” or authoritative role with their sick parent and other older family members. Not only did this dynamic add additional feelings of responsibility, but it also created uncomfortable interpersonal conflicts between EYACs and their family members, as this son demonstrated:

*Honestly, I was pretty angry … Like, “[Mom] if you want something for dinner, I’ll shop for it. We’ll have whatever you like, just don’t put yourself in danger.” It was frustrating to have quite literally quit my job in like a pandemic economy. I’m home to help and to feel like that help was resented and sometimes not appreciated—it definitely hurt.*

*(Son, 22)*

#### 3.3.4. Remembrance Services and Bereavement

After their parents’ death, EYACs reported that pandemic restrictions negatively affected their ability to grieve and memorialize their parent. They described having *delayed or pared down remembrance services* for their parent. Holding smaller funerals and memorial services created additional distress for EYACs, who were torn between preventing COVID-19 spread and wanting to honor their parent with a gathering in their memory. One daughter recalled having to ask people not to attend her father’s memorial service: “We discouraged a lot of people from coming because we didn’t want them to get COVID. And I think quite a few people didn’t show up because they didn’t want to get COVID” (Daughter, 34). Other EYACs and their families opted to delay their parents’ services for months in hopes that pandemic risks and restrictions would subside, as this daughter expressed:

*I expected that we would have done it like in the next week or a week after his death … It definitely sucked … I think we were planning for June or July, because those were his favorite months, and then we’re going to do a nice memorial service. And then that’ll be it.*

*(Daughter, 25)*

In some cases, this delay stunted their grief processing and exacerbated feelings of unfinished business with the deceased parent in the immediate wake of the loss.

In addition to smaller remembrance services, pandemic restrictions also left EYACs unable to grieve with family members, inhibiting communal coping on a historic level. Travel constraints often made it impossible for out-of-town family members and friends to provide in-person support to EYACs. One daughter recalled how difficult it was to be separated from her mother after her father’s death:

*It was hard to support each other because we can’t just visit and hug her or do anything at the time … We don’t know if somebody is going to catch it, so we still have to be really careful. And because of that I think maybe I’d be more mentally stable if COVID wasn’t a thing.*

*(Daughter, 24)*

Another EYAC described feelings of resentment that her mother’s illness and death came at a time that she was unable to receive any family support: “I just kept on thinking like, what if we didn’t have COVID? Like all of my family would have been here. And kind of like, why does she have to get cancer this year?” (Daughter, 30).

## 4. Discussion

The purpose of this study was to examine how the COVID-19 pandemic impacted EYACs who were caring for a parent with advanced cancer. These results provide a preliminary understanding of a specific vulnerable population during a unique historical period. Given the small sample size, these results are best interpreted considering the quantitative and qualitative results together as a combined comprehensive picture of the experiences of pandemic EYACs. Overall, the quantitative results indicated that caring for a parent with advanced cancer during the COVID-19 pandemic resulted in a more demanding and emotionally intense caregiving experience than caring for a parent outside of the pandemic context. This increased intensity manifested as both positive and negative consequences: Pandemic EYACS engaged in more communal coping and found greater benefits in their caregiving experience than pre-pandemic EYACs. In addition, pandemic EYACs experienced higher caregiver strain and emotional distress in comparison to EYACs whose caregiving experience ended before the start of the pandemic. The qualitative results provide insight into why these groups may have differed, illustrating potential sources of pandemic EYACs’ increased distress, caregiver strain, and perceived benefits. EYACs felt the pandemic negatively affected their caregiving efficacy, personal well-being, interpersonal dynamics, bereavement processes, and the quality of their parent’s care. They also described how pandemic-induced changes in employment or schooling increased their day-to-day involvement in their parents’ care and the amount of quality time they were able to spend with their diagnosed parent, which they considered unique benefits of the pandemic.

Seeking and defining personal benefits is one way individuals cope through a challenging experience. Benefit finding is a psychological process by which individuals emphasize the positive outcomes of a difficult experience, interpreting the challenging circumstances as meaningful or useful in retrospect [34]. Benefit finding is associated with long-term positive psychological adjustment and well-being following experiences such as caregiving, even more so when caregivers can also identify the harms of that experience in addition to the benefits [35,36]. In this case, pandemic EYACs reported both higher benefit finding and higher strain and distress, indicating they were constructively weighing the positive and negative aspects of caregiving and psychologically ‘offsetting’ the unique stressors with efforts to seek unique benefits. These findings indicate that although pandemic EYACs experienced intense distress in their caregiving roles, they were also able to tap into this positive coping strategy to adjust in the aftermath of their loss.

As a product of benefit finding coping processes, EYACs reported that their caregiving experiences were often made better by changes in employment and schooling expectations. This may explain why pandemic EYACs reported more communal coping than pre-pandemic EYACs. Global shutdowns, travel restrictions, and shifts to remote work or schooling may have caused caregivers distress, but they also provided opportunities for family members to spend time at home together. This not only allowed EYACs to be more involved in their parent’s care, but it also facilitated their engagement in communal coping with their family members, which has been linked in numerous studies to better adjustment and bereavement outcomes for caregivers [21,22].

In other studies examining the impact of the COVID-19 on caregiving, caregivers also reported pandemic-induced togetherness as a benefit [18,37]. However, increased opportunities for communal coping could have been particularly beneficial for EYACs, as they are more likely than caregivers in other age groups to rely on maladaptive coping strategies and may have needed more support due to the non-normative nature of their caregiving experiences [14,38,39,40,41]. Nevertheless, it is also possible that the context of the pandemic made EYACs more aware of instances where they reached out for support because these activities were “different than normal” (e.g., taking daily family walks to get out of the house; seeking to spend time with friends virtually). This awareness could have led to higher self-ratings of engagement in communal coping.

Pandemic EYACs also reported greater feelings of caregiver strain and more intense negative emotional reactions to their parents’ cancer than pre-pandemic caregivers. This finding could be explained by the additional burdens and pandemic-specific stressors reported in their interviews. EYACs reported struggling with a constant fear that their parent may be exposed to the COVID-19 virus, which mirrored older cancer caregivers’ distress during the pandemic [19], and they described conflicts that arose when their approach to COVID-19 safety differed from that of their parent or other family members. This left pandemic EYACs feeling like they had to “parent their parent.” Adult children who become caregivers for their parents often experience a distressing shift in family roles, suddenly providing care to their parent rather than receiving it [42]. This experience can be heightened for EYACs, whose age and intergenerational relational role may exacerbate the difficulties associated with this shift [41,43].

Additionally, pandemic restrictions forced EYACs and their diagnosed parents out of their daily routines and removed some of the distractions that may have helped them manage their caregiving-related distress (e.g., exercise, working outside the home, and spending time with friends). These losses could have heightened their perception of strain and their negative emotions, particularly in conjunction with the lack of in-person support they were able to receive due to physical distancing and lockdown mandates. Other studies of cancer caregiving during the pandemic also demonstrated that pandemic-induced social isolation and other psychological pressures may have negatively affected caregivers’ well-being [19,44,45]. This impact may be particularly salient for EYACs, as they are at higher risk for developing psychopathology, such as anxiety and depression [14,38,39,40]. The added burdens of isolation and loss of self-care activities along with stress and familial conflicts over COVID-19 safety could explain pandemic EYACs’ increased ratings of caregiving strain and negative emotional reactions in comparison to pre-pandemic EYACs.

The pandemic created significant stress on the global healthcare system, and pandemic EYACs believed that this strain affected the quality of their parent’s cancer care, decreasing crucial in-person medical appointments and adding delays and logistical challenges [1]. Their experiences echoed findings from other studies of cancer patients and their caregivers, who reported feeling like the quality of their care was diminished during the pandemic [4,18]. Although this study did not systematically examine the quality of their parents’ care, EYACs’ perception that their parent did not receive adequate cancer care may have significantly impacted their interpretations of their parent’s medical care experiences and their own insights about the limitations of healthcare systems.

Caregiving for a parent often provides a first opportunity for individuals in this life phase to experience the strengths and limitations of medical systems, process their own reactions to caregiving challenges and loss, and test social dynamics within high-stress scenarios. We speculate that EYACs’ disappointment with medical care options during the pandemic may have cascading implications for their long-term grief adjustment, propensity for post-loss psychological growth, and broader attitudes about healthcare systems [46]. For example, pandemic restrictions limited many of the communication tools palliative care clinicians employ to help family members prepare for bereavement, such as in-person family meetings and end-of-life conversations at the bedside [44]. Their absence, along with the inability to hold remembrance services that provide comfort and opportunities for EYACs to receive support, likely increased their risk of experiencing complicated grief responses [47] and possibly promoted pessimism about navigating future caregiving or health circumstances. Future interventions to support former EYACs should take into account the insights they gleaned from caregiving experiences both within and outside of the context of the pandemic.

EYACs are already a vulnerable caregiving population, but the additional responsibilities and psychological pressures brought on by the COVID-19 pandemic made their risk of emotional distress very high. By providing insight into this under-researched caregiving population, the findings of this study can be used to inform the development of support interventions targeted to EYACs’ unmet needs. Age-specific support is necessary to help this population navigate caregiving and bereavement, both now and in future public health emergencies. Identifying the benefits and negative impacts EYACs’ experienced as a result of the COVID-19 pandemic may be beneficial in reducing their caregiving burden during future pandemic or epidemic events. Clinicians should also be mindful of these findings when working with EYACs who were caregivers during the pandemic. It may be beneficial to offer grief support interventions more commonly associated with early grief (e.g., processing emotions related to loss, identifying ways to honor their deceased parent) because they may not have had those opportunities at the time of their parent’s loss due to COVID-19 restrictions. Additionally, pandemic caregivers may benefit from support groups that include both peers and trained professionals to provide important validation from other EYACs while the professionals clarify misconceptions that may stem for their unique pandemic care contexts.

### Limitations and Future Research

This study was limited in that the data was collected retrospectively; thus, pandemic EYACs likely have more recent memories of their care period than their pre-pandemic counterparts, which could have influenced their reporting of experiences. It is also likely that the caregivers experiencing the most burden may not have had the time or energy to participate in an interview study, meaning these results may not capture the experiences of caregivers who were the most distressed. This study was also limited by its sample size. The quantitative and qualitative results should be interpreted in the context of a small sample size that was made up of primarily White and college-educated people who may not have been as significantly impacted by the pandemic as their minority and/or less educated peers. Sample size and sampling constraints also limited investigation of the associations between contextual factors (e.g., place of death, place of care before death) and outcomes. Additionally, the majority of caregivers included in this study were female, which may have impacted the findings since studies show female caregivers are more likely to have unmet support needs that could also impact their mental distress [48]. Finally, these findings are limited by the lack of information regarding the long-term impacts of these constructs on pandemic EYACs’ continued bereavement, adjustment, and mental health. Future studies should seek to examine these impacts as well as explore these findings with a more racially, ethnically, and socio-economically diverse sample of EYACs.

## 5. Conclusions

Understanding the unique experiences of caregivers during the pandemic is critical to designing resources to support these bereaved caregivers. This may also improve care delivery to EYACs navigating the healthcare system today and in the future. The findings of this study shed light on the additional burden caregivers faced at the height of the COVID-19 pandemic and contribute to the body of work on an under-researched caregiving population. Having this knowledge can help guide clinicians and future researchers to better meet the needs of EYACs and pandemic-affected caregivers.

## Figures and Tables

**Table 1:** Sample Demographics, *n* (%).

		Full Sample	Pandemic Caregivers	Pre-Pandemic Caregivers
Gender				
	Male	14 (35.0%)	6 (33.3%)	8 (36.4%)
	Female	26 (65.0%)	12 (66.7%)	14 (63.6%)
Ethnicity				
	White/Non-Hispanic	30 (75.0%)	13 (72.2%)	17 (77.3%)
	Non-White/Hispanic	10 (25.0%)	5 (27.8%)	5 (22.7%)
Education				
	High school diploma or less	4 (10.0%)	1 (5.6%)	3 (13.6%)
	Some college/trade school	7 (17.5%)	3 (16.7%)	4 (18.2%)
	College graduate	14 (35.0%)	5 (27.8%)	9 (40.9%)
	Post-graduate	15 (37.5%)	9 (50.0%)	6 (27.3%)
Ill Parent				
	Father	26 (65.0%)	11 (61.1%)	15 (68.2%)
	Mother	13 (32.5%)	7 (38.9%)	6 (28.6%)
Relationship Status				
	Married parents	19 (47.5%)	10 (71.4%)	9 (50.0%)
	Single parent	8 (20.0%)	2 (14.3%)	6 (33.3%)
	Remarried/partnered parent	5 (12.5%)	2 (14.3%)	3 (16.7%)
Siblings				
	Has siblings	28 (70.0%)	10 (71.4%)	18 (100.0%)
	Only child	4 (10.0%)	4 (28.6%)	0 (0.0%)
Cancer Type				
	Thoracic	6 (15.0%)	3 (16.7%)	3 (13.6%)
	Brain	5 (12.5%)	3 (16.7%)	2 (9.1%)
	Gastrointestinal	19 (47.5%)	9 (50.0%)	10 (45.5%)
	Other	10 (25.0%)	3 (16.7%)	7 (31.8%)
Age Group				
	Emerging adult (18–25)	14 (35.0%)	7 (38.9%)	7 (31.8%)
	Young adult (26–35)	25 (62.5%)	11 (61.1%)	14 (63.6%)
Age at Diagnosis				
	[in years; M(SD)]	27.03 (4.61)	27.89 (5.25)	26.29 (3.96)
Emerging Adult				
	[IDEA Sum; M(SD)]	2.97 (0.33)	2.94 (0.36)	2.99 (0.31)

**Table 2:** Outcome Descriptives [*M*(*SD*)] and Group Comparisons.

		Full Sample	Pandemic Caregivers	Pre-Pandemic Caregivers	*t*	*g*
Parental Cancer Questionnaire						
Subscale: Caregiver Strain		23.10 (6.67)	24.27 (7.12)	22.11 (6.30)	0.92	0.32 *
Subscale: Emotional Reaction		44.35 (9.26)	45.75 (10.58)	43.29 (8.22)	0.80	0.26 *
Subscale: Perceived Benefits		43.92 (9.70)	45.44 (10.44)	43.12 (9.51)	0.57	0.23 *
Communal Coping		39.97 (7.15)	42.22 (6.55)	38.05 (7.22)	1.88	0.59 **

Note: * = small effect detected, ** = medium effect detected.

## Data Availability

De-identified data from this study are not available in a public archive but will be made available (as allowable according to institutional IRB standards) by emailing the corresponding author.
